# Cardiovascular Care in Africa – Cost Crisis and the Urgent Need for Contextual Health Service Solutions

**DOI:** 10.5334/gh.1259

**Published:** 2023-08-30

**Authors:** Florence Koryo Akumiah, Abdul-Subulr Yakubu, Dzifa Ahadzi, Lawrence Sena Tuglo, Snehasish Mishra, Ranjan K. Mohapatra, Alfred Doku

**Affiliations:** 1 National Cardiothoracic Centre, Korle-Bu Teaching Hospital, Accra, Ghana; 2 Department of Medicine, Tamale Teaching Hospital, Tamale, Ghana; 3 Department of Nutrition and Dietetics, School of Allied Health Sciences, University of Health and Allied Sciences, Ho, Ghana; 4 School of Biotechnology, KIIT Deemed University, Bhubaneswar 751024, India; 5 Department of Chemistry, Government College of Engineering, Keonjhar 758002, Odisha, India; 6 Department of Medicine and Therapeutics, University of Ghana Medical School, Korle-Bu Teaching Hospital, Accra, Ghana; 7 Department of Public and Occupational Health, University of Amsterdam Medical Centre, University of Amsterdam, Netherlands

**Keywords:** cardiovascular diseases, cost crisis, health structures, health service solutions, Africa

## Dear Editor

The 20^th^ century marked a shift in the global disease burden profile with an epidemiological transition from infectious diseases to noncommunicable and degenerative diseases [1]. The rates of transition differed between regions and economic status, with economically backwards nations being saddled with a double burden of infectious and non-communicable diseases. Cardiovascular diseases (CVDs) predominated [2]. Africa encounters such a similar double disease burden. The CVD burden is increasing in Africa, threatening to derail the fragile current health system. The recent global, regional and country-specific economic crises have reduced household incomes, thereby reducing the access to and affordability of guideline-directed care for hypertension, dyslipidemia, diabetes, heart failure, ischemic heart disease, strokes, and the like.

Global statistics show a disproportionately high CVD burden and high morbidity, mortality risk factors and economic load, particularly in the developing world [3]. The CVD toll economically devastates households and governments, especially in low- and middle-income countries, where there is still a competing demand to address the ever-present array of infectious diseases that have worsened after the COVID-19 pandemic [4]. The worsening African healthcare crisis can be viewed from three angles: one, the existing skeletal healthcare infrastructure; two, a lack of trained professionals in handling CVD cases; and three, the increasing incidence of CVD-associated diseases in the backdrop of infectious respiratory diseases such as the recent COVID-19 pandemic.

Globally, billions of dollars are lost in healthcare expenses directly and reduced productivity indirectly from the physical disablement of individuals due to heart failure and other CVDs [5,6]. A significant number of patients with CVDs in sub-Saharan Africa (SSA) pay for CVD care out-of-pocket [4]. Therefore, maintaining patients on guideline-directed medical therapy is challenging, with frequent requests for cheaper therapeutics. Evidence suggests that the very high cost of CVD treatment leads to suboptimal treatment, faulty treatment, and increased CVD-related hospitalization in Africa [7]. Many countries in SSA have fragile economies, making them vulnerable, particularly when there is a downward shift in the global economic climate. Recent cascading global crises due to the COVID-19 pandemic, climate crises, and regional conflicts have aggravated the economic hardships for these countries. The effect further increased the number of already low-income households, hampering the self-financing of cardiovascular care and thereby threatening the achievement of the UN’s Sustainable Development Goal (SDG) by 2030 [8]. Disrupted essential health services and universal health coverage (UHC) may also undermine earlier progress towards achieving the goals.

The 2023 National Daily Minimum Wage was set at GH¢14.88 (~ US$1) in Ghana [9,10]. The current Ghana economy is characterized by a volatile exchange rate and record-high inflation [10]. As a result, the current cost of guideline-directed medical therapy for CVDs, especially for heart failure, easily exceeds more than half of a patient’s annual income in Ghana, stiffening the affordability and contributing to the disease severity that could culminate in increased morbidity and mortality.

Evidence-based preventive measures are needed to curb the growing financial burden imposed by CVDs in Africa and other resource-constrained settings [11]. Health education and health promotion campaigns to empower the population with CVD-preventive information should be pursued as a cost-effective CVD-preventive strategy. Health behaviour at the individual level could be positively impacted by effective communication strategies, avoiding mis- and disinformation. Premordial and primary preventive interventions such as a low salt diet, exercise, reduction in alcohol intake, maintenance of blood pressure, and diabetes control, targeting high-risk groups must be strengthened for cost-effective health management in resource-constrained settings [13]. Robust, systematic, population-based screenings may mitigate catastrophic consequences of CVD through early detection and basic treatment of CVDs, avoiding more expensive treatments later [12]. Healthcare policies are needed that promote better access, affordability and equity through broad-based insurance coverage, improved affordability of drugs and other therapeutics, accessible home-based care, accelerated transition to value-based care and ensuring a trained and professional workforce [7,14,15].

Since the self-financing of healthcare is unsustainable (even if affordable) for an average African, subscribing to health insurance may be encouraged [15]. Bridging the existing coverage gap in this would need political commitment and sustainable financing strategies, exploring alternative pathways for coverage and providing individualized risk-management mechanisms through private payers. While the Ghana National Health Insurance Scheme has dramatically improved access to CVD care, it faces challenges such as frequent unavailability of essential medications and noncoverage of novel evidence-based specialized therapies. The exorbitant over-the-counter price of such therapeutics largely accounts for their exclusion from coverage under health insurance. However, the cost may be met by potential savings from reduced costly hospitalization and therapeutic interventions in the long run [16,17]. By reducing the monopoly, increasing competition, improving access and reducing prices, the local Food and Drug Authority and other appropriate regulatory bodies may concertedly aim to expand the development and market entry of generic and biosimilar drugs. Regulatory barriers limiting the development of generics and biosimilars may be addressed. The possibility of fostering the development of advanced manufacturing platforms that reduce costs without compromising on improved quality and reliability could also be explored. The government’s efforts to achieve UHC for CVD care with the ultimate aim of attaining SDG goals seem elusive without urgent solutions.
